# Implementation of a novel living-donor kidney transplant preoperative checklist within the electronic medical record: a pilot study

**DOI:** 10.1186/s13037-015-0074-5

**Published:** 2015-08-19

**Authors:** Bradley C. Gill, Hans C. Arora, Hannah R. Kerr, Stuart M. Flechner, Courtney D. Ellis, David A. Goldfarb

**Affiliations:** Glickman Urological and Kidney Institute, Cleveland Clinic, Mail Stop Q10-1, 9500 Euclid Ave, Cleveland, OH 44195 USA; Department of Biomedical Engineering, Cleveland Clinic, Cleveland, OH USA; Cleveland Clinic Lerner College of Medicine, Case Western Reserve University, Cleveland, OH USA

## Abstract

**Background:**

Checklist utilization in surgery has contributed to improved patient safety and reduced numbers of preventable complications. A living-donor kidney transplant (LDKT) preoperative checklist embedded within electronic medical record (EMR) was developed to enhance patient safety and prevent “never” events including: unexpected donor-recipient blood (ABO) incompatibility, positive (XM) cross match, infectious disease transmission, or procurement of an anatomically inappropriate allograft. Review of the initial 2 years of checklist utilization was performed.

**Findings:**

This safety instrument operates by facilitating critical review and referencing of source documentation to confirm ABO, XM, infectious risk, and organ anatomy compatibility. It was met with high compliance rates and no “never events” have occurred following its inception. The checklist is readily available in the EMR and is accessible by all members of the LDKT recipient healthcare team.

**Conclusions:**

Checklist utilization was associated with zero LDKT “never event” occurrences. Surgeons felt the checklist was easy to use.

## Findings

### Introduction

Living donor kidney transplantation is accepted worldwide as the preferred form of renal replacement. Recently, there has been enhanced attention to LDKT per reports of preventable disease transmission and organ incompatibility [1–6]. Transmissible disease in solid organ transplantation is rare. A study by the Organ Procurement and Transplantation Network (OPTN) with the United Network for Organ Sharing (UNOS) determined that in 2007 up to 5 malignancies and 12 infections were transmitted with solid organ transplants [3]. The UNOS website notes 28,366 solid organ transplants were completed that year, which translates into a maximum 0.056 % rate of transmissible disease. As such, several aspects of LDKT require critical attention to make the process as safe as possible and prevent ‘never events’ from occurring. These elements include attestation of ABO compatibility, knowledge of infectious transmission risk, attestation of XM compatibility, and assurance of appropriate organ anatomy. Lack of knowledge in any of these domains could lead to serious morbidity that is otherwise preventable.

Surgical checklists have been used successfully to improve safety. A number of studies attest to the effectiveness of checklists in accomplishing this [7–11]. To date, no prior publication describes the prevention of ‘never events’ in LDKT due to safety checklist implementation. The aim of this project was to design an EMR-embedded safety checklist to prevent ‘never events’ in LDKT and evaluate its use.

## Methods

**Fig. 1:**
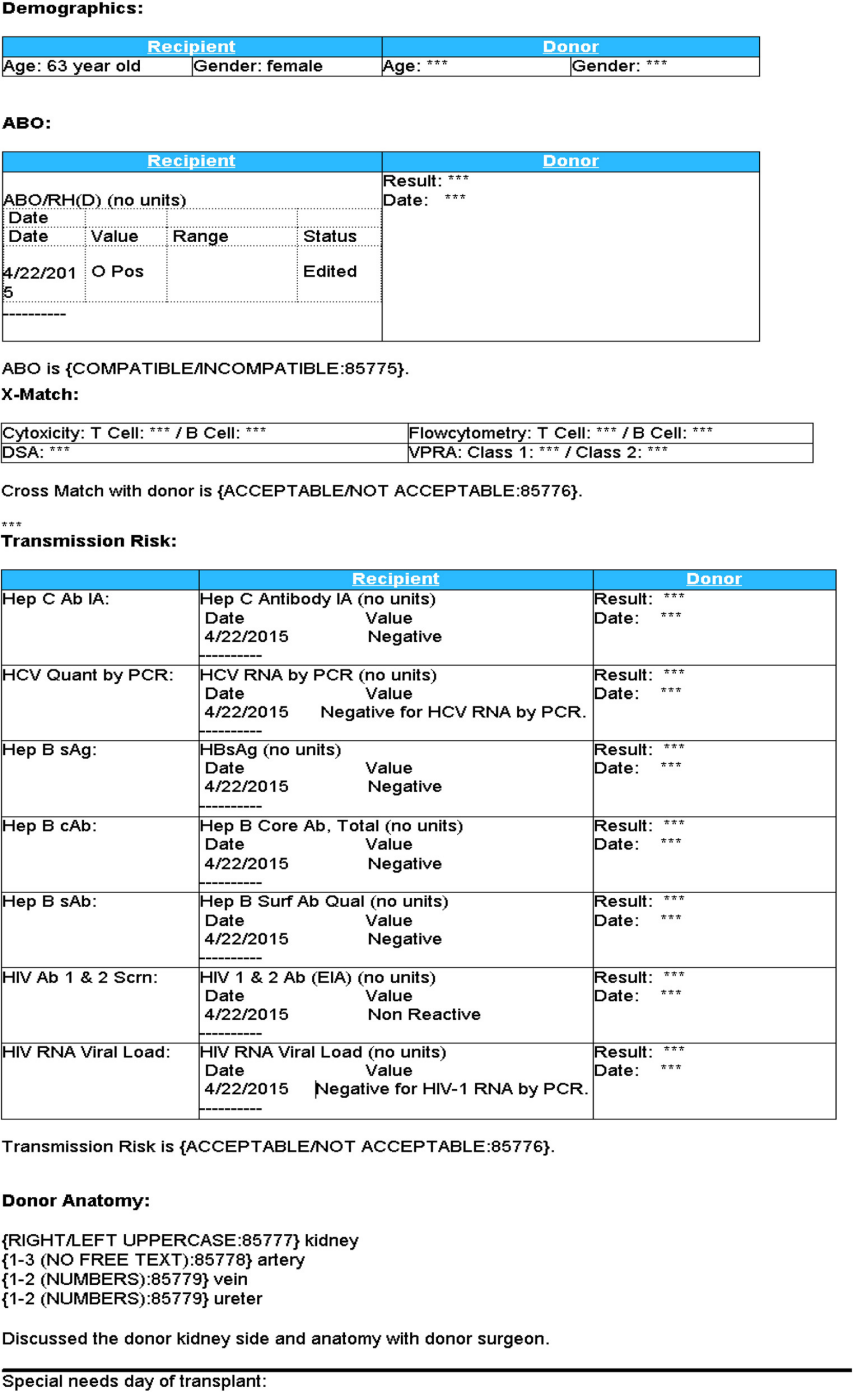
Depiction of the Electronic Medical Record checklist template

The checklist was created and embedded in the health system EMR (EPIC, Verona, Wisconsin). It is a separate encounter, easily created by the recipient surgeon, and is viewable by anyone accessing the patient record. The checklist (Fig. 1) contains 5 information domains: demographics, ABO, XM, infectious disease status, and donor organ laterality with anatomic details. Demographic and basic laboratory data from the recipient is populated automatically from the EMR into the left column of the checklist, preventing transcription error when consolidating the information. Compatibility information, specifically, cytotoxicity XM testing, flow XM testing, donor specific antibody status, and calculated/virtual panel reactive antibody (VPRA) are entered manually by the recipient surgeon.

Donor information is reviewed directly from source documentation in the EMR and/or outside records. Donor information is then manually inserted into the right column of the checklist by the recipient surgeon, and includes demographics, ABO, and infectious risk. No donor information is auto-populated into the checklist. This ensures the surgeon critically reviews this information at the time of data entry, enabling any abnormalities to be identified and addressed.

At the conclusion of the ABO, XM, and infectious risk sections there is a surgeon attestation statement, which confirms compatibility (ABO, XM) and safety (infectious disease transmission risk) of the transplant. The organ anatomy section includes kidney laterality, the number of arteries, veins, and ureters, as well as an attestation confirming discussion with the donor surgeon. Donor anatomy is determined from available images.

The responsibility for checklist creation rests with the recipient surgeon. Checklists are completed prior to transplantation and as soon as possible once all data is available. Based upon checklist results, additional or repeat testing can be ordered as necessary, or surgery may be cancelled if indicated. The checklists are reviewed by the surgical team in the preoperative setting prior to any anesthetic or surgical intervention. This study evaluates checklist completion rates and effectiveness over the first 27 months of its use. Local institutional review board approval (study 13–1538) was obtained.

## Results

Recipient checklists from September 2011 deployment through December 2013 were retrospectively reviewed. A total of 157 LDKT checklists were obtained. Mean recipient age was 48.4 years with 56.6 % male and 43.5 % female (Table 1). Average body mass index was 26.6 kg/m^2^ for the group. Checklist completion rate was 87.9 % across five transplant surgeons.

**Table 1:** Group demographics and overall completion rate

Measure	Mean or Percentage
Recipient Age (Years)	48.4
Recipient Body Mass Index (kg/m^2^)	26.6
Recipient Sex (Male)	56.6 %
Checklist Completion Rate (Overall)	87.9 %

All completed checklists contained recipient ABO, while 5.1 % lacked donor information. However, a total of 98.6 % noted ABO compatibility. Similarly, XM information was omitted in 7.2 % of checklists (up to 18.1 % lacked calculated VPRA data) but overall XM was acceptable in 93.5 % with one erroneously noting incompatibility (Table 2).

**Table 2:** Blood type and immunologic information

Checklist Measure	*N* = 157
Missing Blood Type Data	5.1 %
Blood Type Marked Compatible	98.6 %
Missing Cross Match Data	7.2 %
Cross Match Marked Compatible	93.5 %

Infectious risk was completely documented in all but 6.5 % of checklists while 98.5 % were deemed acceptable (Table 3). Anatomic details were present in all but 3.6 % of checklists. Only 1 (paired donor organ from another hospital) form did not confirm donor and recipient surgeon discussion.

**Table 3:** Infectious risk and anatomic details

Checklist Measure	*N* = 157
Missing Infectious Risk Data	6.52 %
Infectious Risk Marked Acceptable	98.5 %
Missing Anatomic Data	3.6 %
Anatomy Discussed with Donor Surgeon	99.4 %

One transplant was cancelled the day before surgery per inadequate infectious screening identified by the checklist. No hepatitis C nucleic acid testing result was available, so the case was postponed 2 weeks. There were no “never events” (Table 4). Overall, transplants surgeons found the checklists easy to use and quick to complete.

**Table 4:** Outcomes during checklist use

Checklist Measure	*N* = 157
“Never Events”	0.0 %
Surgery Cancellation	0.0 %
Surgery Delay or Rescheduling	0.66 %

## Discussion

Living donor transplantation carries risks beyond those routinely associated with surgery. Significant morbidity can be attributed to these events, which include ABO mismatch, infectious transmission, XM incompatibility, and inappropriate organ anatomy [1, 5, 6]. However, all are preventable with accurate pre-procedural knowledge. Many areas in healthcare and other industries utilize checklists to reduce rates of errors and catastrophic events [7–11]. This served as motivation for the development of the LDKT checklist.

An EMR-based safety checklist was developed, which is easy to use and helps prevent LDKT ‘never events’ from occurring. Recipient information from the EMR is automatically loaded into the checklist. Donor information is manually entered into the tool directly from source documentation, fostering critical review of each entry by the surgeon. Additionally, the tool requires safety attestations in multiple domains by the recipient surgeon. It also enables source documentation information to be centralized into a single location that is readily accessible to all transplant team members .

While the advantages of this checklist have been discussed, limitations also exist. Manual entry of donor data directly from source documentation does foster critical review, however it is subject to human error and inaccurate data transcription. Likewise, it is possible that critical consideration is not given to abnormal values that may present – another illustration of the human factor. Either situation could potentially result in adverse events. Incorporating review of the completed checklist by another surgeon, versus his or her completion of a separate checklist for comparison, may reduce the chance of erroneous checklist data, but again, would not eliminate it due to human nature.

Documented transmission of donor-derived infectious disease and/or malignancy highlight the types of incidents such a checklist can prevent [12, 2, 13–19]. A study of pre-operative checklist use in living donor liver transplantation produced positive results, however recipient outcomes were not thoroughly investigated [20]. Overall, the LDKT checklist presented here was met with good utilization by the transplant surgeons and was deemed easy to use. It provided a final checkpoint prior to transplantation once all donor and recipient information was known.

Since LDKT checklist implementation, no “never events” have occurred. One transplant was deferred due to missing infectious risk information. This eliminated any harm that may have been conferred to the organ recipient. It also showed that the surgical team must remain committed, and if needed, make difficult decisions based upon checklist results. Consideration is being given to implementing a similar tool for cadaveric kidney transplantation, however, in a much more acute timeframe, the utility and feasibility of completing such a checklist is unclear.

Incomplete compliance was noted in various checklist domains. In some instances, compatibility and/or infectious transmission risk were known by the surgeon and deemed acceptable, but test results to support the attestation were not entered. This lack of completeness is problematic, as the medical record is a legal document where lacking documentation can be presumed to signify tests were not completed. Short of 100 % compliance, effectiveness of the checklist is compromised. Promoting compliance will require continued surgeon education and an emphasis on checklist importance.

A recent institutional initiative called for the development of care paths to standardize patient management, ensure quality, and control costs. A renal transplantation care path was created and requires use of the LDKT checklist. Furthermore, as part of a quality initiative, a departmental quality officer now monitors compliance and notifies surgeons of any deficiencies with checklist completion to foster improvement. The checklist is updated periodically. It was recently expanded to include viral nucleic acid testing, West Nile virus testing, and Strongyloides testing, if indicated, per updated institutional and UNOS guidelines.

Strengths of this study include the review of all patients scheduled for LDKT, which identifies all cases regardless of whether a checklist was completed or not, as opposed to querying the EMR for the presence of a checklist. Another strength is that the study occurred in a high-volume, multi-surgeon transplant group. This allows the usage patterns of different individuals to be averaged together, potentially making the data more generalizable. Limitations of the study are inherent to its retrospective nature.

## Conclusions

An EMR-based safety checklist for LDKT was successfully designed and implemented. Retrospective review of its utilization showed good initial compliance. During use of the LDKT checklist, no “never events” occurred. The checklist is easy to use and can be completed quickly.

## Abbreviations

EMR: Electronic medical record
LDKT: Living donor kidney transplant
ABO: Blood type
XM: Cross match
VPRA: Calculated / virtual panel reactive antibody
